# *De novo* nucleotide biosynthesis and its dynamic regulation are crucial for systemic infection by extraintestinal *Escherichia coli*

**DOI:** 10.1371/journal.ppat.1013889

**Published:** 2026-01-26

**Authors:** Chu Zhang, Yi Yang, Shuang Wang, Yuying Liu, Xianglin Zhao, Fan Yin, Yawen Hu, Ying Xue, Zixuan Bu, Shaowen Li, Chen Tan, Rui Zhou, Qi Huang

**Affiliations:** 1 National Key Laboratory of Agricultural Microbiology, Hubei Hongshan Laboratory, College of Veterinary Medicine, Huazhong Agricultural University, Wuhan, China; 2 Institute of Modern Biology, State Key Laboratory of Pharmaceutical Biotechnology, Nanjing University, Nanjing, China; 3 International Research Center for Animal Disease, Ministry of Science and Technology of China, Wuhan, China; 4 Cooperative Innovation Center for Sustainable Pig Production, College of Veterinary Medicine, Huazhong Agricultural University, Wuhan, China; Ruhr-Universität Bochum: Ruhr-Universitat Bochum, GERMANY

## Abstract

Extraintestinal *Escherichia coli* (ExPEC) proliferates rapidly in the bloodstream, causing life-threatening systemic infections with high mortality rates. Understanding how ExPEC adapts to the blood environment is essential for elucidating its pathogenesis and identifying potential antimicrobial targets. Using transposon mutagenesis-based high-throughput screening, we systematically identified genes critical for ExPEC growth in serum. Our findings demonstrate that *de novo* nucleotide biosynthesis genes are indispensable for serum proliferation, and animal infection assays further confirm their vital role in ExPEC virulence. Genetic disruption of this pathway did not affect bacterial stress response or adhesion capacity but severely impaired ExPEC’s intracellular survival in macrophages. Transcriptomic profiling combined with a luminescent reporter system revealed significant upregulation of *de novo* nucleotide biosynthesis genes during both serum incubation and systemic infection. This upregulation was mediated by the transcriptional repressor PurR, and could be inhibited by purine supplementation. Furthermore, enhanced *in vivo* fitness was observed for the *purR* deletion strain, whereas the opposite was seen for the *purR* overexpression strain, indicating a crucial role for PurR in ExPEC pathogenesis. Quantitative analyses showed that serum incubation significantly depletes intracellular purine pools in ExPEC. Electrophoretic mobility shift assays (EMSA) demonstrated that specific purines modulate PurR-DNA binding affinity. These results suggest that PurR acts as a sensor of intracellular purine concentration changes, regulating *de novo* nucleotide biosynthesis genes to facilitate host environment adaptation. This study reveals the essential role of *de novo* nucleotide biosynthesis in ExPEC virulence and describes a pathogenesis mechanism involving nucleotide metabolism regulation to overcome nutritional immunity, offering a foundation for developing therapies against systemic ExPEC infections.

## Author summary

Extraintestinal pathogenic *Escherichia coli* (ExPEC) exhibits a remarkable ability to proliferate in the bloodstream, enabling rapid dissemination throughout the host. Identifying genetic determinants that support ExPEC growth in blood is crucial for understanding its pathogenic mechanisms and discovering potential therapeutic targets. In this study, we identified the critical role of *de novo* nucleotide biosynthesis genes in serum growth and systemic infection by ExPEC. Moreover, we demonstrated that the *de novo* nucleotide biosynthesis pathway is significantly upregulated during incubation in serum or during systemic infection. PurR was identified as a key repressor of this pathway and contributed to ExPEC pathogenesis. We further provide biochemical evidence showing that purine levels modulate the DNA-binding affinity of PurR. This mechanism allows PurR to function as a molecular sensor of nucleotide availability, tuning *de novo* nucleotide biosynthesis gene expression to overcome nutritional constraints within the host. These findings provide valuable insights into ExPEC pathogenesis and highlight promising therapeutic targets.

## Introduction

Extraintestinal pathogenic *Escherichia coli* (ExPEC) poses a significant global health threat as a versatile bacterial pathogen capable of causing severe or even life-threatening diseases in both humans and animals [1,2]. The clinical spectrum of ExPEC infections is broad, with primary manifestations including urinary tract infections (UTIs), meningitis with particular concerns in neonates, and sepsis, which has a high mortality risk [3–5]. Of particular concern is that the level of antimicrobial resistance is very high, and vaccine development is a big challenge due to high genomic diversity [2,6–8]. The World Health Organization (WHO) has recognized ExPEC as one of the global priority endemic pathogens requiring urgent control measures [9].

One of the most significant characteristics of ExPEC is its remarkable capacity for rapid proliferation within host environments. Unlike intestinal pathogenic *E. coli* strains that primarily rely on enterotoxins, for example, Shiga toxins [10], heat-stable and/or heat-labile toxins [11], etc., as major virulence determinants, ExPEC exhibits distinct pathogenic mechanisms, with host survival and immune evasion playing central roles, although toxins have also been reported to be involved in the pathogenesis of ExPEC [12,13]. A well-studied example is the iron acquisition system, in which a large-scale genomic association study revealed a major role of the iron capture systems in the virulence of ExPEC [14]. Also, the iron regulator Fur serves as the key regulator of the ExPEC, controlling expression of a substantial amount of genes in response to the host environment, for example, in the serum [15]. The ability to utilize a variety of amino acids and small peptides has also been revealed to be associated with the pathogenicity of uropathogenic *Escherichia coli* (UPEC) to cause UTIs [16].

Nucleotide biosynthesis is essential for all organisms, which produces building blocks required for many key cellular processes, including DNA replication, energy metabolism, cell signaling, etc. [17]. There are two pathways that bacteria use to synthesize nucleotides: the *de novo* biosynthesis pathway and the salvage pathway. The *de novo* pathway synthesizes nucleotides from precursor molecules, including ribose-5-phosphate and some amino acids [18]. The salvage pathway catalyzes pre-existing purine or pyrimidine bases and nucleosides to synthesize nucleotides [18]. Nucleotide biosynthesis has been reported to play important roles in the pathogenesis of several bacterial pathogens. For example, disrupting the *de novo* purine biosynthesis constrains intracellular replication of UPEC [19]. In *Staphylococcus aureus*, the *de novo* purine biosynthesis but not the salvage pathway is reported to be essential for the virulence [20].

PurR is a key transcriptional repressor of nucleotide biosynthesis in both Gram-negative and Gram-positive bacteria [21,22]. Biochemical studies revealed that most purine biosynthesis and some of the pyrimidine biosynthesis genes are directly regulated by PurR [21]. Recent studies have provided evidence linking PurR to the virulence of pathogenic bacteria. For example, *purR* mutation results in hypervirulence in *S. aureus,* which is attributed to its regulatory role in *de novo* purine biosynthesis [22]. This indicates the important role of transcriptional regulation of nucleotide biosynthesis for bacterial pathogenesis.

ExPEC can lead to serious systemic infections. Although research has identified nucleotide biosynthesis as a contributing factor in the pathogenesis of certain ExPEC strains, such as UPEC [19,23,24], the specific mechanisms by which this contributes to ExPEC virulence, as well as the dynamic regulation of this metabolic pathway during systemic infections, are not well understood.

In this study, transposon mutagenesis screening identified the *de novo* nucleotide biosynthesis as essential for serum growth of ExPEC. The specific contribution of this pathway to the pathogenesis was investigated. Moreover, our data demonstrated significant upregulation of the pathway during infection and revealed that PurR functions as a metabolic sensor, modulating bacterial adaptation to host environments through purine concentration-dependent regulation of nucleotide biosynthesis.

## Results

### Genome-wide screening reveals the essential role of *de novo* nucleotide biosynthesis genes in ExEPC serum survival

**Fig 1:**
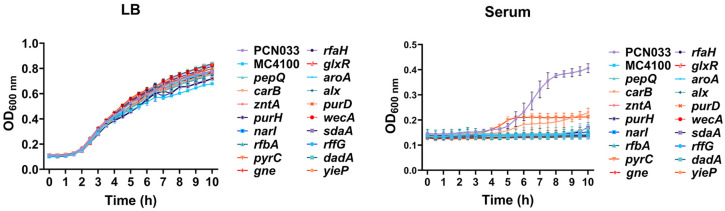
Growth curves of Tn mutants. Overnight cultures of each indicated strain were inoculated 1:100 into LB medium (left panel) or fresh mouse serum (right panel). OD_600 nm_ was measured every 0.5 hours. The ExPEC PCN033 strain served as a positive control with robust growth in serum, while the *E. coli* MC4100 strain was used as a negative control that was unable to grow in serum.

In comparison to the commensal intestinal *E. coli* MC4100 strain, the ExPEC PCN033 (WT) strain demonstrated robust growth in mouse serum (S1 Fig), consistent with its strong ability to proliferate within the host. A genome-wide screening was conducted using transposon (Tn) mutagenesis to identify genes required for serum growth of ExPEC. A total of 4500 Tn mutants were generated using our previously established Tn mutagenesis system [25] and tested for their growth in mouse serum. Compared to the WT strain, eighteen mutants were identified that displayed significantly inhibited growth in the serum (Table 1). Growth curves were further measured with these Tn mutants, revealing that in contrast to the WT strain, these mutants exhibited retarded growth in serum, although they showed a similar growth pattern in LB medium (Fig 1). Notably, genes involved in *de novo* nucleotide biosynthesis, including *purH*, *purD*, *pyrC*, and *carB*, were significantly enriched among those identified in the Tn screening. Additionally, several other genes were identified, primarily related to cell envelope biogenesis (*rffG*, *wecA*, *rfbA*, *gne*), amino acid transport and metabolism (*aroA*, *dadA*, *sdaA*, *pepQ*), energy production and conversion (*narI*), lipid transport and metabolism (*glxR*), and transcription regulation (*rfaH* and *yieP*).

**Table 1:** Essential genes for ExPEC growth in serum.

Gene	Description	Growth ratio^a^	P value	COG group	COG function
***purH***	bifunctional AICAR transformylase/IMP cyclohydrolase	0.343	4.8e-3	F	Nucleotide transport and metabolism
***purD***	phosphoribosylamine--glycine ligase	0.351	9e-4	F	
***pyrC***	Derived by automated computational analysis using gene prediction method: Protein Homology	0.524	4.8e-3	F	
***carB***	carbamoyl-phosphate synthase large subunit	0.562	2.4e-3	F	
***aroA***	3-phosphoshikimate 1-carboxyvinyltransferase	0.329	2.8e-3	E	Amino acid transport and metabolism
***dadA***	D-amino acid dehydrogenase	0.352	5e-4	E	
***sdaA***	L-serine ammonia-lyase	0.419	2.9e-2	E	
***pepQ***	Xaa-Pro dipeptidase	0.412	1.4e-3	E	
***rffG***	dTDP-glucose 4,6-dehydratase	0.376	4.4e-3	M	Cell wall/membrane/envelope biogenesis
***wecA***	UDP-N-acetylglucosamine--undecaprenyl-phosphate N-acetylglucosaminephosphotransferase	0.333	5.1e-3	M	
***rfbA***	mannose-1-phosphate guanylyltransferase/mannose-6-phosphate isomerase	0.329	5.3e-3	M	
***gne***	N-acetyl-alpha-D-glucosaminyl-diphospho-ditrans, octacis-undecaprenol 4-epimerase	0.333	1.6e-3	M	
***yieP***	FadR family transcriptional regulator	0.346	5.2e-3	K	Transcription
***rfaH***	transcription/translation regulatory transformer protein RfaH	0.331	4.2e-3	K	
***alx***	TerC family protein	0.359	4e-4	P	Inorganic ion transport and metabolism
***zntA***	Zn(II)/Cd(II)/Pb(II) translocating P-type ATPase ZntA	0.356	5.2e-3	P	
***narI***	respiratory nitrate reductase subunit gamma	0.343	5.7e-3	C	Energy production and conversion
***glxR***	2-hydroxy-3-oxopropionate reductase	0.355	7e-4	I	Lipid transport and metabolism

^a^The growth ratio indicates the relative growth of the Tn mutant versus the wild-type (WT) strain in serum. This ratio is calculated by dividing the OD_600 nm_ value of the Tn mutant (OD_Tn_) by the OD_600 nm_ value of the WT strain (OD_WT_) following a ten-hour incubation in serum. The P value was determined using Student’s t-test by comparing the final OD_600 nm_ of the WT strain and the Tn mutant in serum.

### *The de novo* nucleotide biosynthesis pathway is critical for the virulence of ExPEC

**Fig 2:**
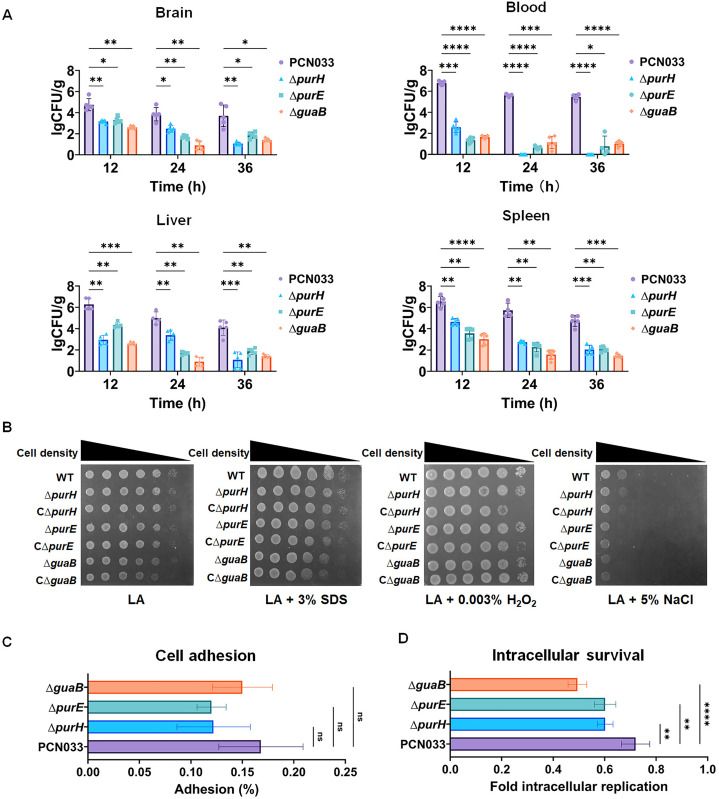
*De novo* nucleotide biosynthesis genes are critical for ExPEC virulence. **A. Animal infection assays.** Mice were intraperitoneally injected with 2 × 10⁵ CFU of mid-log phase cultures of each indicated strain. Bacterial burdens in tissues were quantified at each indicated time point. **B. *In vitro* stress response assays.** Mid-log phase cultures of each strain were diluted to 1 × 10^6^ CFU/ml, and 5 μL of each cell suspension was spotted onto the indicated agar plates, followed by incubation at 37°C. **C. Cell adhesion assay.** PK-15 cells were infected with mid-log phase cultures of ExPEC PCN033 and its mutants at a multiplicity of infection (MOI) of 10:1 and incubated at 37°C for one hour. The cell culture was then washed twice with sterile PBS, followed by the addition of sterile water and incubation at 4°C for one hour to lyse cells. The lysate was serially diluted and plated on LB agar for bacterial enumeration. **D. Intracellular survival assay.** Bacterial cultures were mixed with RAW264.7 cells at an MOI of 10:1 and incubated at 37°C for one hour. After washing twice with sterile PBS, unphagocytized bacteria were killed by incubation with PBS containing 50 μg/ml chloramphenicol. Cells were then cultured in fresh DMEM. After two hours, macrophages were lysed as described above for bacterial enumeration. Statistical significance was assessed using one-way ANOVA. Significance levels were defined as ns (not significant), P < 0.05 (*), P < 0.01 (**), P < 0.001 (***), and P < 0.0001 (****).

Following the identification of multiple *de novo* nucleotide biosynthesis genes *via* Tn screening, we evaluated the contribution of this metabolic pathway to ExPEC virulence using a murine infection model. *purH,* which was identified in the Tn screening, and two other *de novo* purine biosynthesis genes, *purE* and *guaB*, were selected, and their deletion mutants and complemented strains were constructed. Growth assays showed that Δ*purH*, Δ*purE*, and Δ*guaB* were unable to grow in the serum, and complementation of each gene restored the growth, confirming the critical role of the *de novo* nucleotide biosynthesis pathway for growth in the serum (S2 Fig). The mouse infection model was used to compare the virulence of the mutants with that of the WT strain. It was shown that Δ*purH*, Δ*purE*, and Δ*guaB* strains were rapidly cleared in the indicated organ tissues while the WT strain still showed high load at 12, 24, and 36 hours post-infection (hpi), suggesting that disrupting the *de novo* purine biosynthesis pathway significantly attenuated the virulence of ExEPC (Fig 2A). It was further investigated whether deleting the *de novo* nucleotide biosynthesis impaired the ability of stress response of ExPEC by testing their growth in the presence of 0.003% H_2_O_2_ (oxidative stress), 3% SDS (envelop stress), or 5% NaCl (hyperosmotic stress). The results showed that Δ*purH*, Δ*purE*, and Δ*guaB* exhibited comparable growth with the WT strain (Fig 2B), suggesting that the attenuated virulence was not attributed to the stress response ability. Cell adhesion experiments were performed, which showed that the deletion of *purH*, *purE*, or *guaB* did not affect the cell adhesion (Fig 2C). In contrast, Δ*purH*, Δ*purE*, and Δ*guaB* exhibited defective intracellular survival within macrophages (Fig 2D). These data suggest that disruption of the *de novo* nucleotide biosynthesis pathway significantly attenuated the virulence of ExPEC, which was attributed to the impaired growth in the serum and within macrophages.

### The expression of *de novo* nucleotide biosynthesis genes is significantly upregulated when incubated in serum and during ExPEC infection

**Fig 3:**
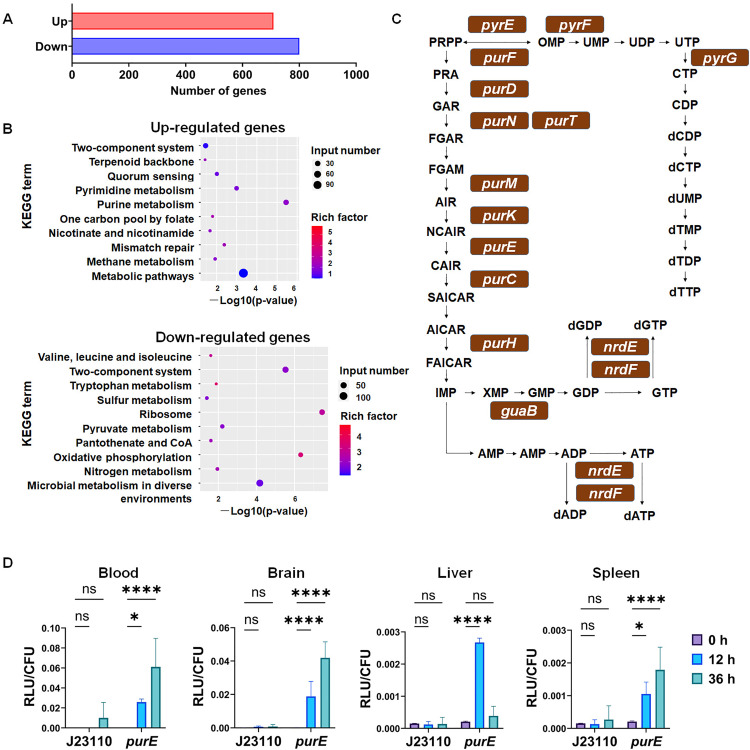
*De novo* nucleotide biosynthesis genes show significant upregulation during serum incubation and infection. **A. Statistics of up- and down-regulated genes in ExPEC after serum incubation. B. KEGG pathway enrichment analysis of differentially expressed genes (DEGs).** The DEGs identified in transcriptomic analysis were subjected to KEGG pathway enrichment, with the top 10 enriched pathways shown. **C. Schematic diagram of upregulated genes in the *de novo* nucleotide biosynthesis pathway.** Upregulated genes are marked with brown boxes. **D. Expression of the *purE* gene during mouse infection.** ExPEC PCN033 carrying either pCDF-J23110-Lux or pCDF-purR-Lux was used to infect mice. Mice were humanely euthanized at indicated time points, followed by tissue collection and homogenization for luminescence measurement and bacterial load quantification. Statistical significance was assessed using one-way ANOVA. Significance levels were defined as ns (not significant) and P < 0.0001 (****).

Transcriptomics analysis was conducted to identify differentially expressed genes (DEGs) in ExPEC when cultured in serum compared with LB medium. RNA-sequencing (RNA-seq) revealed that compared with incubation in LB medium, one-hour incubation in fresh serum induced upregulation of 800 genes and downregulation of 709 genes of ExPEC PCN033 (Fig 3A). KEGG enrichment analysis of DEGs demonstrated that the top 10 enriched pathways for upregulated genes included: Two-component system, Terpenoid backbone biosynthesis, Quorum sensing, Pyrimidine metabolism, Purine metabolism, One carbon pool by folate, Nicotinate and nicotinamide metabolism, Mismatch repair, Methane metabolism, and Metabolic pathways. The top ten enriched pathways for downregulated genes comprised: Valine, leucine, and isoleucine degradation, Two-component system, Tryptophan metabolism, Sulfur metabolism, Ribosome, Pyruvate metabolism, Pantothenate and CoA biosynthesis, Oxidative phosphorylation, Nitrogen metabolism, and Microbial metabolism in diverse environments (Fig 3B). Notably, genes involved in both purine and pyrimidine nucleotide biosynthesis showed significant enrichment. Pathway mapping revealed substantial involvement of these genes in *de novo* nucleotide biosynthesis (Fig 3C). To verify whether *de novo* nucleotide biosynthesis genes were similarly upregulated during systemic infection, luminescent reporter strains were generated by fusing the promoter regions of *purE* with the *luxCDABE* coding sequence. Using constitutive promoter J23110 as a control, significant upregulation of *purE* gene was observed at 12 hpi and 36 hpi (Fig 3D). These findings collectively demonstrate significant activation of the *de novo* nucleotide biosynthesis pathway during ExPEC infection.

### Supplementation of purines in serum inhibited the upregulation of *de novo* purine biosynthesis genes in ExPEC

**Fig 4:**
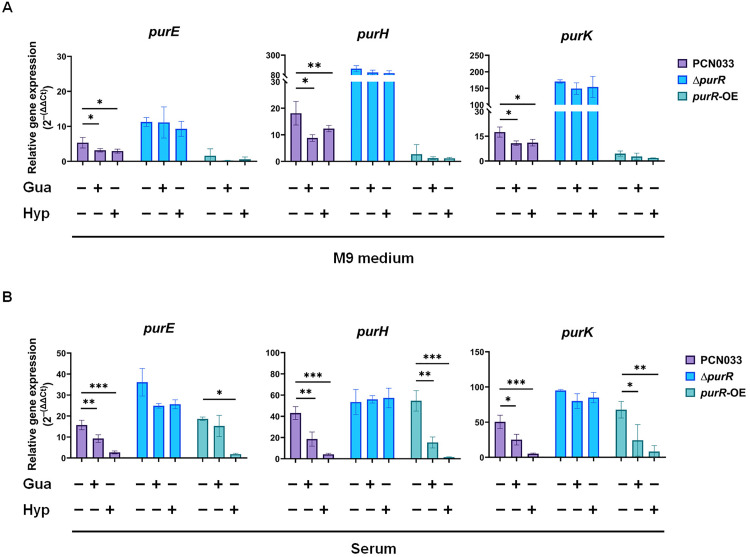
Expression analysis of *de novo* purine biosynthesis genes in ExPEC under different conditions and strains. Gene expression was determined using RT-qPCR with WT, Δ*purR*, and *purR*-OE strains in M9 medium **(A)** or serum **(B)**, with or without supplementation of 0.1 mM guanine (Gua) or hypoxanthine (Hyp). Statistical significance was assessed using one-way ANOVA. Significance levels were defined as ns (not significant), P < 0.05 (*), P < 0.01 (**), and P < 0.001 (***).

It was tested whether the upregulation of *de novo* purine biosynthesis genes was caused by the scarcity of free purines in serum. The expression of these genes was determined in M9 medium (purine-free) and serum, respectively, with or without supplementation of additional purines. As shown in Fig 4 (purple columns), when ExPEC PCN033 cells were incubated in M9 medium or in serum, significant upregulation of the *de novo* purine biosynthesis genes *purE*, *purH*, and *purK* was observed. In contrast, when additional guanine or hypoxanthine was supplemented to the M9 medium or serum, the upregulation of these genes was significantly inhibited. These results indicated that the *de novo* purine biosynthesis genes were upregulated in response to limited environmental availability of free purines.

### PurR is a transcriptional repressor that regulates the expression of *de novo* purine biosynthesis genes and plays a role in the pathogenesis of ExPEC

PurR has been previously identified as a transcriptional regulator of nucleotide biosynthesis. To investigate its regulatory role in ExPEC, we constructed a *purR* deletion strain (Δ*purR)* and an overexpression strain (*purR*-OE). Western blot analysis confirmed the overexpression of PurR in the latter strain (S3 Fig). Since the addition of exogenous purines inhibited the upregulation of *de novo* purine biosynthesis genes when ExPEC cells were incubated in M9 medium or serum (Fig 4), it was next tested whether this regulation was mediated by PurR by testing the expression of the purine biosynthesis genes in Δ*purR* and *purR*-OE strains, with or without supplementation of additional purines. The results showed that in both M9 medium and serum, deletion of *purR* significantly upregulated the expression of purine biosynthesis genes (Figs 4A & 4B). In contrast to the observation that the addition of extra purines inhibited the upregulation of these genes in the WT strain, supplementing guanine or hypoxanthine to serum or M9 medium did not inhibit the upregulation in the Δ*purR* strain (Fig 4A & 4B, blue columns), suggesting that upregulation of purine biosynthesis genes in response to purine scarcity was mediated by PurR. In addition, expression analysis in *purR*-OE strain showed that *purR* overexpression resulted in repression of purine biosynthesis genes and supplementation of purines could further enhance the suppressing effect (Fig 4A & 4B, teal columns).

**Fig 5:**
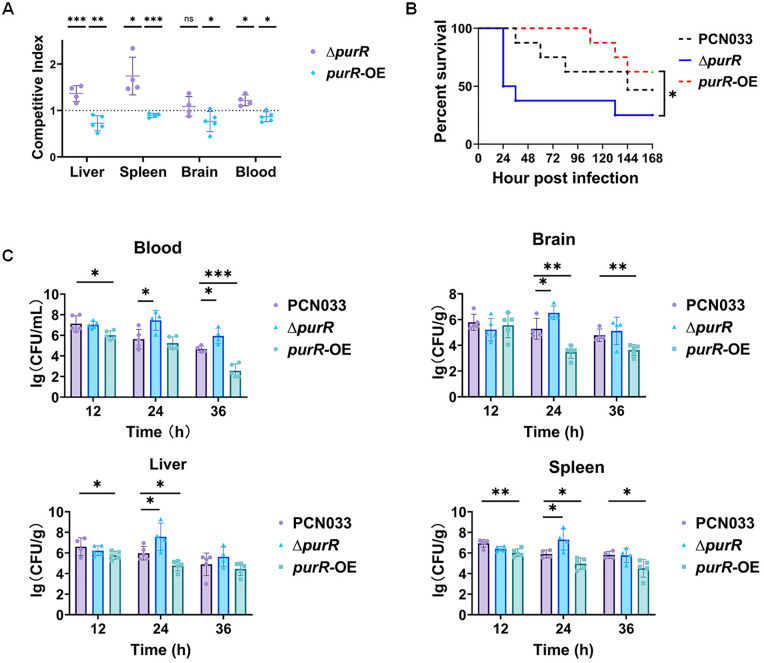
PurR contributes to *in vivo* fitness of ExPEC. **A. Competitive infection assays.** Cells of Δ*purR* or *purR*-OE strains were mixed with ExPEC PCN033 at a 1:1 ratio, and the mixture containing a total of 2 × 10^5^ CFU was intraperitoneally injected into mice. At twelve hours post-infection, mice were humanely euthanized, and bacterial burden in tissue samples was quantified for each strain. The competitive index (CI) was calculated by dividing the ratio of the two strains’ loads in the tissues by their corresponding ratio in the inoculum. Statistical analysis was performed using Student’s t-test by comparing the CI of the indicated strain (Δ*purR* or *purR*-OE) with that of the WT strain. Significance levels were defined as ns (not significant), P < 0.05 (*), P < 0.01 (**), and P < 0.001 (***). **B. Mouse survival assays.** 6 × 10^5^ CFU cells of the mid-log phase WT, Δ*purR*, or *purR*-OE strains were used to intraperitoneally inject mice, respectively. Survival of each group was recorded every day. n = 8. Statistical differences were examined using the Gehan-Breslow-Wilcoxon test, and * indicates P < 0.05. **C. *In vivo* colonization assays.** 2 × 10^5^ CFU cells of the mid-log phase WT, Δ*purR*, or *purR*-OE strains were used to infect mice *via* intraperitoneal injection, respectively. Mice from each group were euthanized at 12, 24, and 36 hpi, and the blood, brain, liver, and spleen samples were collected. The tissues were homogenized, diluted with sterile saline, and plated on LA plates. Colonies were counted after overnight incubation. Statistical significance was assessed using one-way ANOVA. Significance levels were defined as ns (not significant), P < 0.05 (*), P < 0.01 (**), and P < 0.001 (***).

Given the critical role of *de novo* nucleotide biosynthesis genes in ExPEC pathogenesis, it was next examined whether their regulator PurR was also involved in virulence. Using competitive infection assays, we compared the relative *in vivo* fitness of the Δ*purR* and *purR*-OE strains against the WT strain. The results showed that Δ*purR* strain demonstrated significantly greater competitive fitness in the liver, spleen, and blood (competitive index > 1, P < 0.05) (Fig 5A). In contrast, the *purR*-OE strain exhibited markedly reduced fitness in all four tissues tested (competitive index < 1, P < 0.05) (Fig 5A). To further delineate the role of *purR* in the virulence of ExPEC PCN033, we conducted individual mouse infection experiments with each of the WT, Δ*purR*, and *purR*-OE strains using identical infection doses. Mouse survival assay results showed that following a lethal dose infection (6 × 10^5^ CFU, intraperitoneal injection), mice infected with the Δ*purR* strain exhibited reduced survival compared to WT-infection mice (Fig 5B). In contrast, infection with the *purR*-OE strain resulted in a trend to increased survival relative to the WT strain (Fig 5B). The percent survival showed a statistically significant difference between the Δ*purR* and *purR*-OE groups (P = 0.026). Bacterial colonization was further assessed by measuring bacterial burdens in various organ tissues at specific timepoints post-infection following infection using a sub-lethal dose (2 × 10^5^ CFU, intraperitoneal injection). The results showed that at 24 hpi, mice infected with the Δ*purR* strain exhibited significantly higher bacterial loads in the blood, brain, liver, and spleen compared with those infected with the WT strain (Fig 5C). However, the *purR*-OE strain displayed significantly reduced colonization in the blood (12 and 36 hpi), brain (36 hpi), liver (12 hpi), and spleen (12, 24, and 36 hpi). Collectively, these results demonstrate that PurR plays a crucial role in ExPEC pathogenesis.

### Purines modulate the binding of PurR to its target genes

**Fig 6:**
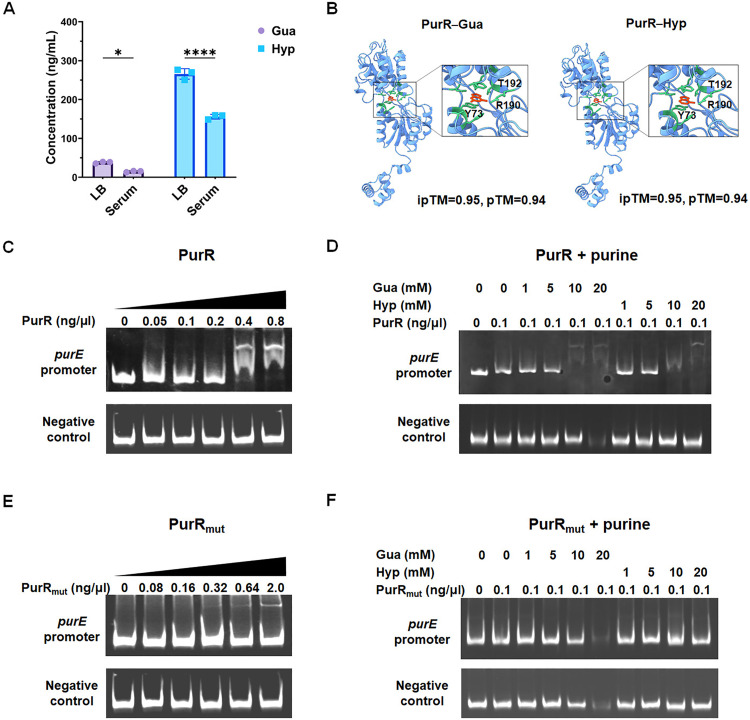
Purine precursors influence the binding affinity of PurR to its target. **A. Quantification of intracellular purine precursors.** ExPEC PCN033 was cultured to mid-log phase in either mouse serum or LB medium. Intracellular metabolites were extracted, and the concentrations of guanine and hypoxanthine were quantified using liquid chromatography-tandem mass spectrometry (LC-MS/MS). **B. Molecular docking of PurR with guanine and hypoxanthine.** The three-dimensional structures of PurR-guanine and PurR-hypoxanthine complexes, along with their ipTM and pTM scores, were predicted using a locally implemented AlphaFold3 server. The PurR protein is depicted in blue, while the ligands (guanine or hypoxanthine) are shown in red. The residues in the binding pocket are shown in green. **C & E. Electrophoretic mobility shift assay (EMSA) analysis of PurR binding to the** *purE* **promoter region.** Purified His-tagged PurR (**C**) or PurR_mut_ (**E**) at various concentrations was incubated with the *purE* promoter DNA fragment in binding buffer at room temperature for 30 minutes. The reaction mixtures were separated on 6% native polyacrylamide gels, stained with GelRed, and visualized using a gel imaging system. **D & F. Effect of guanine and hypoxanthine on PurR-DNA binding.** PurR (**D**) or PurR_mut_ (**F**) at a non-binding concentration (0.1 ng/μL) was incubated with the *purE* promoter region in the presence or absence of varying concentrations of guanine (Gua) or hypoxanthine (Hyp). The binding reactions were electrophoresed on 6% native polyacrylamide gels, stained with GelRed, and analyzed using a gel imaging system.

It was next investigated how the *de novo* purine biosynthesis genes of ExPEC are dynamically regulated during infection. Quantitative measurement of intracellular guanine and hypoxanthine concentrations in ExPEC using ultra performance liquid chromatography (UPLC) revealed significantly lower levels of both purines when the bacteria were incubated in serum compared to LB medium (Fig 6A). To study the interaction between PurR and guanine or hypoxanthine, we performed protein-ligand docking using the state-of-the-art deep-learning approach AlphaFold3. The predicted interface TM (ipTM) scores for both the PurR–guanine and PurR–hypoxanthine complexes were 0.95, indicating high-confidence interactions. (Fig 6B). EMSA analysis of PurR binding to the promoter region of its target gene *purE* showed concentration-dependent band shifts, while no band shifts were observed when incubating with an unrelated control fragment, confirming PurR-*purE* promoter interaction (Fig 6C). Notably, at suboptimal PurR concentrations that did not show *purE* promoter binding, the addition of guanine or hypoxanthine enhanced PurR-*purE* promoter complex formation (Fig 6D). To further confirm the influence of purine on target binding, PurR_mut_, a PurR variant harboring point mutations (Y73A, R190A, T192A) in its substrate-binding pocket, was purified. EMSA results showed that PurR_mut_ retained the ability to bind the *purE* promoter. However, unlike wild-type PurR, the addition of guanine or hypoxanthine did not enhance binding. These results demonstrate that guanine and hypoxanthine increase the binding affinity of PurR to its target DNA.

## Discussion

ExPEC is well-known for its rapid proliferation within the host, leading to systemic infections with high mortality rates. The bloodstream serves as a critical defense barrier, eliminating invading pathogens through immune mechanisms (including immune cells and the complement system) and nutrient limitation [26]. A hallmark of ExPEC pathogenesis is its ability to overcome these restrictions and multiply in the blood, facilitating dissemination throughout the host [27–29]. Therefore, identifying genes essential for serum growth is crucial for elucidating the mechanism of ExPEC pathogenesis and discovering novel antimicrobial drug targets. Through using Tn screening, 18 serum growth-related genes were identified. In addition to the genes involved in nucleotide biosynthesis, *rffG*, *wecA*, *rfbA*, and *gne* form another functionally enriched group involved in lipopolysaccharides or extracellular polysaccharides biosynthesis. Disruption of these genes may compromise cell envelope integrity. Studies have reported that impairment of bacterial surface polysaccharides reduced serum survival by decreasing complement killing resistance as well as pathogenicity [26,28,30]. Several amino acid metabolism genes were also identified, including *aroA*, *dadA*, *sdaA*, and *pepQ*. Among these, *aroA* is responsible for the synthesis of aromatic amino acids, which is a well-characterized virulence-related gene of several bacterial pathogens and has been used as a live vaccine candidate gene [31]. The others are involved in amino acid utilization, with the deficiency of *dadA* or *sdaA* having been reported to influence cell wall synthesis, potentially impairing the stress resistance [32,33]. *alx* and *zntA* encode two ion transporters related to manganese and zinc transport, respectively [34,35]. Disruption of the gene impairs serum growth, indicating the importance of maintaining cytoplasmic ion pools for the *in vivo* fitness of ExPEC.

Our results demonstrate that *de novo* nucleotide biosynthesis genes are not only critical for serum growth but also essential for the full virulence of ExPEC in an animal model, confirming their crucial role in ExPEC pathogenesis, which aligns with previous studies [19,36]. We further revealed that disruption of the *de novo* purine biosynthesis pathway did not affect the *in vitro* stress response capability nor adhesion to host cells, but impaired proliferation in host cells (Fig 2). These findings indicate that *de novo* nucleotide biosynthesis enables ExPEC to overcome the nutrient limitations within the host, thereby promoting its growth *in vivo*. Notably, disruption of these genes did not impair ExPEC growth in rich medium but nearly abolished the growth within the host. Therefore, they present promising drug targets for developing antimicrobials with minimal selective pressure, as these inhibitors would only exert antimicrobial activity under specific conditions, such as during host infection. A recent study identified a small-molecule inhibitor targeting the *de novo* purine biosynthesis protein PurF in *Mycobacterium tuberculosis,* which demonstrated potent bactericidal activity both *in vitro* and *in vivo* [37].

One limitation of this study is that although both purine and pyrimidine biosynthesis pathways were identified in our Tn mutagenesis screening, the focus remained primarily on the purine biosynthesis pathway. In fact, as shown in Table 1, two *de novo* pyrimidine biosynthesis genes, *pyrC* and *carB*, were identified as critical for ExPEC growth in serum, suggesting their potential role in bacterial pathogenesis. This finding aligns with a previous study that also identified the requirement of both purine and pyrimidine biosynthesis for *E. coli* growth in human blood [38]. We further examined the regulatory mechanism of purine biosynthesis mediated by PurR. However, the regulation of pyrimidine biosynthesis was not dissected in this study, although our RNA-seq data also showed significant upregulation of this pathway upon incubation in serum (Fig 3). Previous studies have suggested that the regulatory mechanisms governing pyrimidine biosynthesis are often far more intricate, involving complex strategies such as attenuation control, transcription start site switching, and reiterative transcription initiation [39]. The detailed regulation of pyrimidine biosynthesis in response to the host environment, and its exact role in bacterial pathogenesis, represents a significant and complex area of research that warrants dedicated future investigation.

Apart from the critical role of the *de novo* pathway in nucleotide biosynthesis, the vital role of salvage pathways has also been previously reported for the pathogenesis of UPEC [40]. Specifically, Ma *et al.* revealed that salvage pathways for purine synthesis could support the optimal growth of UPEC in urine. In our Tn screening, no genes involved in the salvage pathways of nucleotide biosynthesis were identified. This discrepancy may be explained by differences in nutrient availability between the two host niches: urine is relatively rich in free purines and nucleobases but poor in carbon sources, whereas serum contains abundant glucose and amino acids yet extremely low levels of free nucleotides. Consequently, ExPEC appears to adaptively switch its nucleotide acquisition strategy by favoring salvage in urine and activating *de novo* synthesis in blood. This reflects the metabolic flexibility of ExPEC in adapting to the host environment, which contributes to its broad disease spectrum.

Another important finding of this study is that *de novo* nucleotide biosynthesis genes show significant upregulation during serum incubation or ExPEC infection (Fig 3). Notably, the upregulation of purine biosynthesis is regulated by the transcriptional repressor PurR. Deletion of *purR* relieved this suppression, enhancing the *in vivo* fitness of ExPEC, suggesting that dynamic regulation of nucleotide biosynthesis plays a crucial role in ExPEC pathogenesis. Further data demonstrate that serum incubation caused decreased concentration of purines in ExPEC cells, and biochemical analysis showed that purine levels influence PurR’s binding affinity to its target DNA.

ExPEC employs both the *de novo* pathway and the salvage pathway for nucleotide biosynthesis. Disruption of the *de novo* pathway does not affect growth in rich medium (Fig 1), indicating that when the free nucleotides are abundant, ExPEC primarily uses the salvage pathway, making the *de novo* pathway non-essential. However, when environmental free nucleotides are scarce, such as during serum incubation or host infection, intracellular purine levels in bacteria decline. This reduces the binding of purines (e.g., guanine and hypoxanthine) to the repressor PurR, weakening PurR’s interaction with its target genes, primarily those involved in nucleotide biosynthesis. As a result, PurR-mediated repression of the *de novo* nucleotide biosynthesis pathway is relieved, leading to its upregulation. This allows ExPEC to synthesize nucleotides using the relatively abundant sugars and amino acids available in blood, thereby circumventing extracellular nucleotide limitation and supporting robust proliferation in the bloodstream. Thus, our findings reveal a pathogenic mechanism whereby ExPEC dynamically regulates nucleotide biosynthesis in response to host nucleotide limitation to facilitate its infection.

## Materials and methods

### Ethics statement

All animal housing and experimental procedures were approved by the Institutional Animal Care and Use Committee of the Animal Center of Huazhong Agricultural University (approval number HZAUMO-2025–0243).

### Bacterial strains and culture conditions

All *E. coli* strains used in this study are listed in S1 Table. The wild-type ExPEC strain PCN033 is a highly virulent and multidrug-resistant strain, and its murine model of systemic infection has been well established [25,41]. Gene deletion mutants of ExPEC PCN033 were constructed by allelic exchange using the suicide vector pRE112 as described previously [42]. Gene complementation was achieved by introducing a pCDF-derived recombinant plasmid containing the indicated gene, including its promoter region, into the respective deletion mutant. PurR overexpression was achieved by introducing the plasmid pCDF-J23110-PurRhis into the ExPEC PCN033 strain to constitutively express PurR, resulting in the *purR*-OE strain. All the *E. coli* strains were grown in LB or LB-agar (LA) medium at 37°C with shaking, and antibiotics were used when appropriate during bacterial culture.

### Transposon-directed screening of genes essential for serum survival

Tn mutants of ExPEC PCN033 were generated using a *Himar*1 mariner-based transposon mutagenesis approach established in our previous study [25]. To screen Tn mutants required for growth in the serum, the mid-log phase bacterial culture of the individual Tn mutant was diluted 100-fold (~5 × 10⁴ CFU) into fresh mouse serum (purchased from Oumarsi (Shanghai) Biotechnology Co., Ltd., China; Cat# OMHD-E0069), followed by incubation at 37°C for ten hours. ExPEC PCN033 was used as a positive control that showed robust growth, and *E. coli* MC4100, a commensal strain, was used as a negative control that did not grow in the serum. Optical density at the wavelength of 600 nm (OD_600 nm_) was measured to evaluate bacterial growth, and the growth ratio was calculated as OD_600 nm_ of Tn mutant divided by the OD_600 nm_ of ExPEC PCN033 strain. Mutants showing significantly reduced growth in serum were selected for Tn insertion site identification using a two-round nested PCR followed by Sanger DNA sequencing as described previously [25]. Clusters of Orthologous Groups (COG) annotation of the genes was performed using eggNOG-mapper [43].

### RNA-sequencing and data analysis

To determine the transcriptional response of ExPEC upon transition from rich medium to serum, ExPEC PCN033 cells were cultured in LB medium to the mid-log phase. The cells were subsequently washed three times with sterile saline and inoculated into fresh mouse serum, giving an initial concentration of 10^8^ CFU/ml, followed by incubation for one hour at 37°C. Both groups contained three biological replicates. The cells were then harvested, and total RNA was extracted using the RNeasy Kit (Cat# 4992858, Tiangen) with on-column DNase I treatment. RNA quality was assessed using an Agilent 2100 Bioanalyzer. rRNA was depleted using the Ribo-Zero kit (Cat# 20040529, Illumina), and strand-specific libraries were prepared for sequencing on an Illumina NovaSeq platform. Raw reads were trimmed and mapped to the ExPEC PCN033 reference genome using HISAT2 [44]. Differentially expressed genes were identified using DESeq2 [45] with thresholds of |log₂ fold change| > 1 and FDR < 0.05. Kyoto Encyclopedia of Genes and Genomes (KEGG) annotation and enrichment were analyzed using clusterProfiler [46].

### Animal experiments

Animal infection experiments were conducted using four-week-old female specific pathogen-free (SPF) Kunming (KM) mice. In the mouse survival assay, a lethal dose of 6 × 10^5^ CFU of mid-log phase ExPEC PCN033 or its derivative strains was used to infect mice *via* intraperitoneal injection. Survival was monitored daily. To assess bacterial colonization *in vivo*, mice were intraperitoneally injected with 2 × 10^5^ CFU of ExPEC PCN033 or its derivatives. At 12, 24, and 36 hours post-infection (hpi), the mice were euthanized, and organ samples (including liver, spleen, blood, and brain) were collected. The tissues were homogenized in sterile saline, serially diluted, and then plated onto LB agar for CFU enumeration. Competitive infection assays were performed to compare the *in vivo* fitness differences between the two strains, as previously described [25]. Briefly, a 1:1 mixture of mid-log phase cells of two strains containing a total of 2 × 10^5^ CFU was intraperitoneally injected into mice. The mice were euthanized at 12 hpi, followed by bacterial burden quantification of each strain in the tissue samples. The competitive index was calculated by dividing the ratio of the two strains’ loads in the tissues by their corresponding ratio in the inoculum.

### Cell adhesion and intracellular survival assay

To assess cell adhesion, cells of the porcine kidney cell line PK-15 were seeded in 24-well plates and cultured in Dulbecco’s modified Eagle’s medium supplemented with 10% heat-inactivated fetal bovine serum. The cells were infected with the mid-log phase cells of EXPEC PCN033 and its mutant derivatives in 24-well plates at a multiplicity of infection (MOI) of 10:1 and incubated at 37°C with 5% CO_2_ for one hour. The cell culture was then washed twice with sterile PBS, and sterile water was added, which was incubated at 4°C for 1 hour for cell lysis. The lysate was serially diluted and applied to LB agar plates for bacterial counting [25]. For the intracellular survival assay, the bacterial cells were mixed with RAW264.7 cells with an MOI of 10:1, followed by incubation at 37°C for one hour. Then, the cells were washed twice with sterile PBS, and the unphagocytized bacterial cells were killed by incubation with PBS containing chloramphenicol (final concentration 50 μg/ml). The cells were continued to be cultured in fresh DMEM. After two hours of incubation, macrophages were lysed in the same way as above for bacterial counting [19,25].

### Quantitative Real-Time PCR (RT-qPCR) analysis

The mid-log phase cells of EXPEC PCN033 or its derivatives were incubated at 37°C under different conditions (serum or M9 medium, with or without supplementing guanine or hypoxanthine) for one hour. Subsequently, the bacterial cells were harvested by centrifugation, and total RNA was extracted using the RNeasy Kit (Cat# 4992858, Tiangen) with on-column DNase I treatment. RNA purity and concentration were assessed spectrophotometrically with A260/A280 ratios of 1.8 – 2.0. cDNA was synthesized from 1 μg RNA using the PrimeScript RT Reagent Kit (Cat# RR036A, Takara, Japan). Quantitative PCR was performed in triplicate using qPCR Mix (Cat# RR430A, Takara, Japan) on a CFX96 Real-Time PCR System (Bio-Rad, USA). The housekeeping *gapA* gene served as the internal reference, and relative gene expression was calculated *via* the 2^−ΔΔCt^ method.

### Protein-ligand docking with Alphafold3

Docking of PurR of ExPEC with guanine or hypoxanthine was performed with a locally installed Alphafold3 [47]. The predicted complex structure was visualized using ChimeraX [48].

### Quantitation of intracellular concentrations of purines

ExPEC PCN033 was incubated in mouse serum or LB to the mid-log phase. Bacterial cells were harvested, washed twice with ice-cold PBS. Intracellular metabolites were extracted using 80% methanol at -80°C and dried under vacuum. Metabolite concentrations were quantified using liquid chromatography-tandem mass spectrometry (LC-MS/MS) with an Agilent 1290 UHPLC coupled to a 6470 triple quadrupole mass spectrometer.

### Electrophoretic mobility shift assay

Electrophoretic mobility shift assay (EMSA) was performed as described previously [49]. His-tagged PurR or PurR containing point mutations was expressed using the pET28a vector system and purified using nickel-ion affinity chromatography. The purified PurR protein with varied concentrations was incubated with the DNA fragment of the promoter region of the target genes or an unrelated fragment as a negative control in binding buffer (0.3 M NaCl and 0.05 M NaH_2_PO_4_) at room temperature for 30 min in the absence or presence of different concentrations of guanine or hypoxanthine. Binding reactions were resolved on 6% native polyacrylamide gels, followed by staining with Gelred and detection using a gel imaging system.

### Statistical analysis

Data are presented as the mean ± standard error of the mean (SEM). Student’s *t*-test was used to determine statistical significance between two groups, while one-way analysis of variance (ANOVA) was used for comparisons among multiple groups. Survival curves were analyzed using the Gehan-Breslow-Wilcoxon test. All statistical analyses were performed using GraphPad Prism 8 software. P values < 0.05, < 0.01, < 0.001, and < 0.0001 were considered statistically significant and are denoted by *, **, ***, and ****, respectively.

## Supporting information

S1 Fig Growth curves of ExPEC and commensal *E. coli* in serum. Overnight cultures of ExPEC PCN033 strain and *E. coli* MC4100 strain were inoculated at a 1:100 ratio into fresh mouse serum. The OD_600 nm_ was measured hourly.
(TIF)

S2 Fig Growth curves of deletion mutants and their complemented strains of *de novo* nucleotide biosynthesis genes in serum. Overnight cultures of each strain were inoculated 1:100 into fresh mouse serum. OD_600 nm_ was measured every two hours.
(TIF)

S3 Fig Western blotting analysis of PurR expression. ExPEC strain PCN033 or PCN033 carrying pCDF-J23110-PurRhis plasmid (*purR*-OE) was grown to the mid-log phase in LB medium. Cells were harvested, and the whole cell lysate and lysate supernatant were prepared. The samples were subjected to SDS-PAGE followed by Western blotting analysis using an anti-His monoclonal antibody as the primary antibody.
(TIF)

S1 Table Bacterial strains used in this study. (DOCX)
