# Supplementary figures and images for: De novo nucleotide biosynthesis and its dynamic regulation are crucial for systemic infection by extraintestinal Escherichia coli
Source: PLoS Pathog. 2026 Jan 26;22(1):e1013889. doi: 10.1371/journal.ppat.1013889 (PMC12858057; doi:10.1371/journal.ppat.1013889)

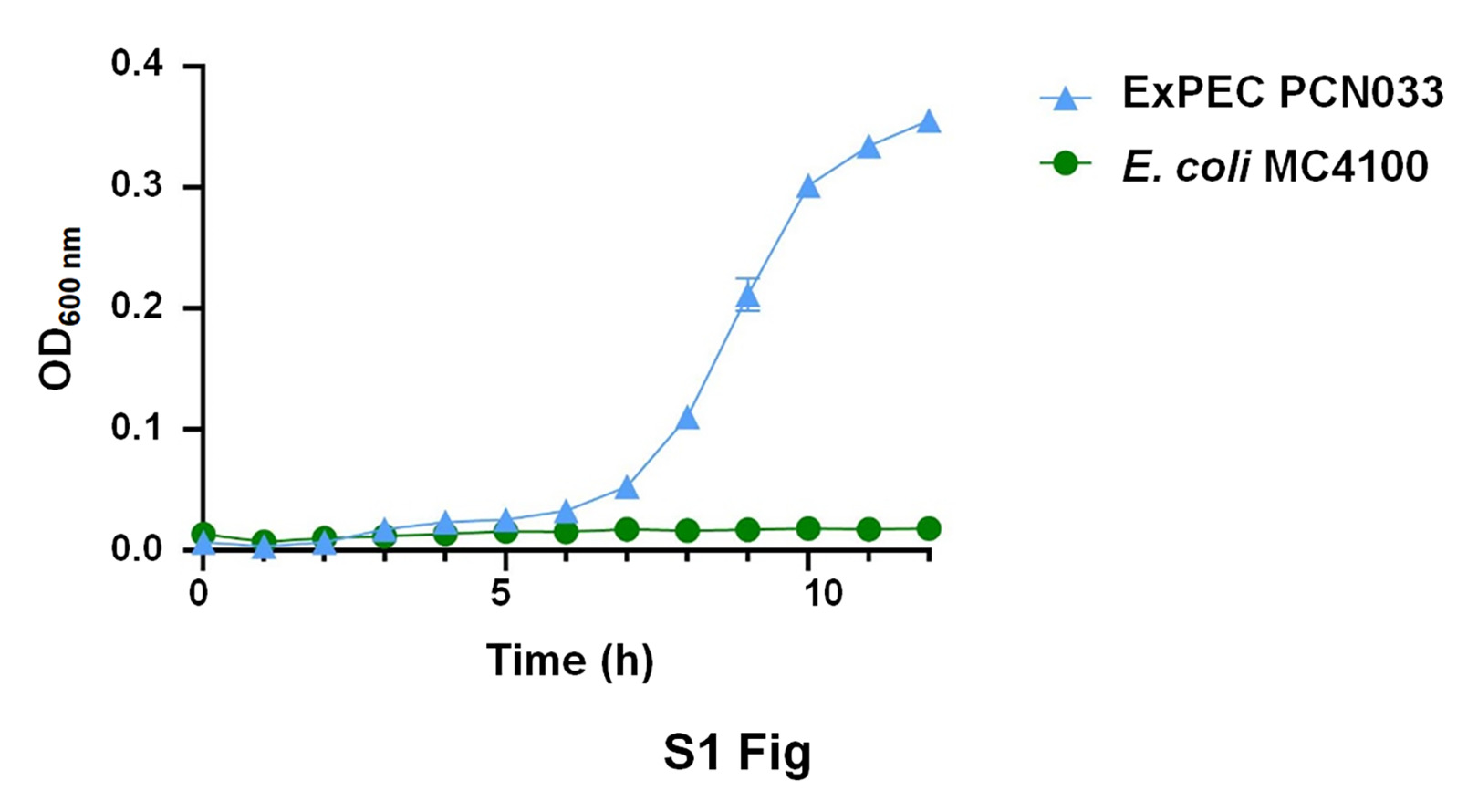

Supplement: S1 Fig — Overnight cultures of ExPEC PCN033 strain and E. coli MC4100 strain were inoculated at a 1:100 ratio into fresh mouse serum. The OD600 nm was measured hourly. (TIF) [file ppat.1013889.s001.tif]

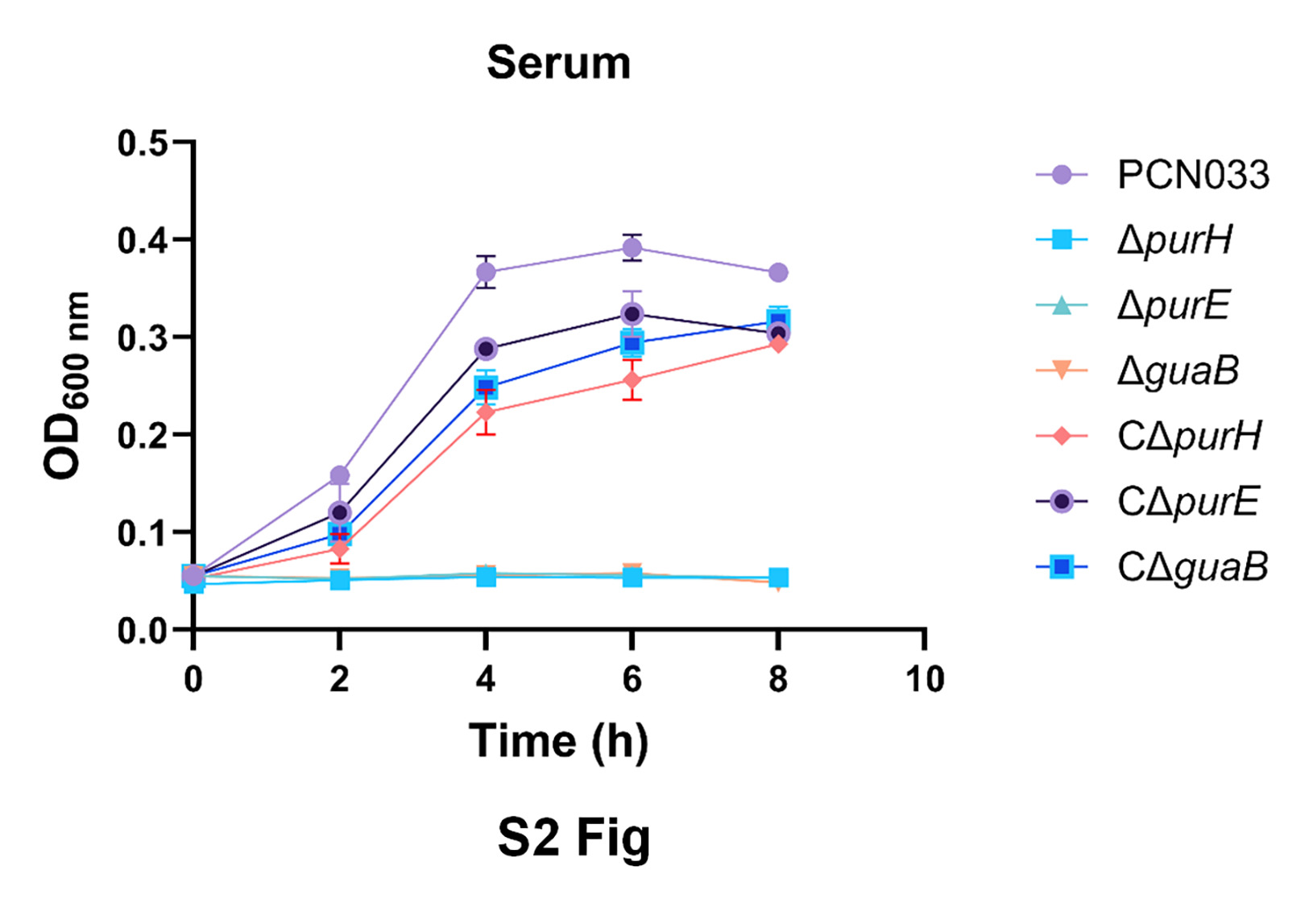

Supplement: S2 Fig — Overnight cultures of each strain were inoculated 1:100 into fresh mouse serum. OD600 nm was measured every two hours. (TIF) [file ppat.1013889.s002.tif]

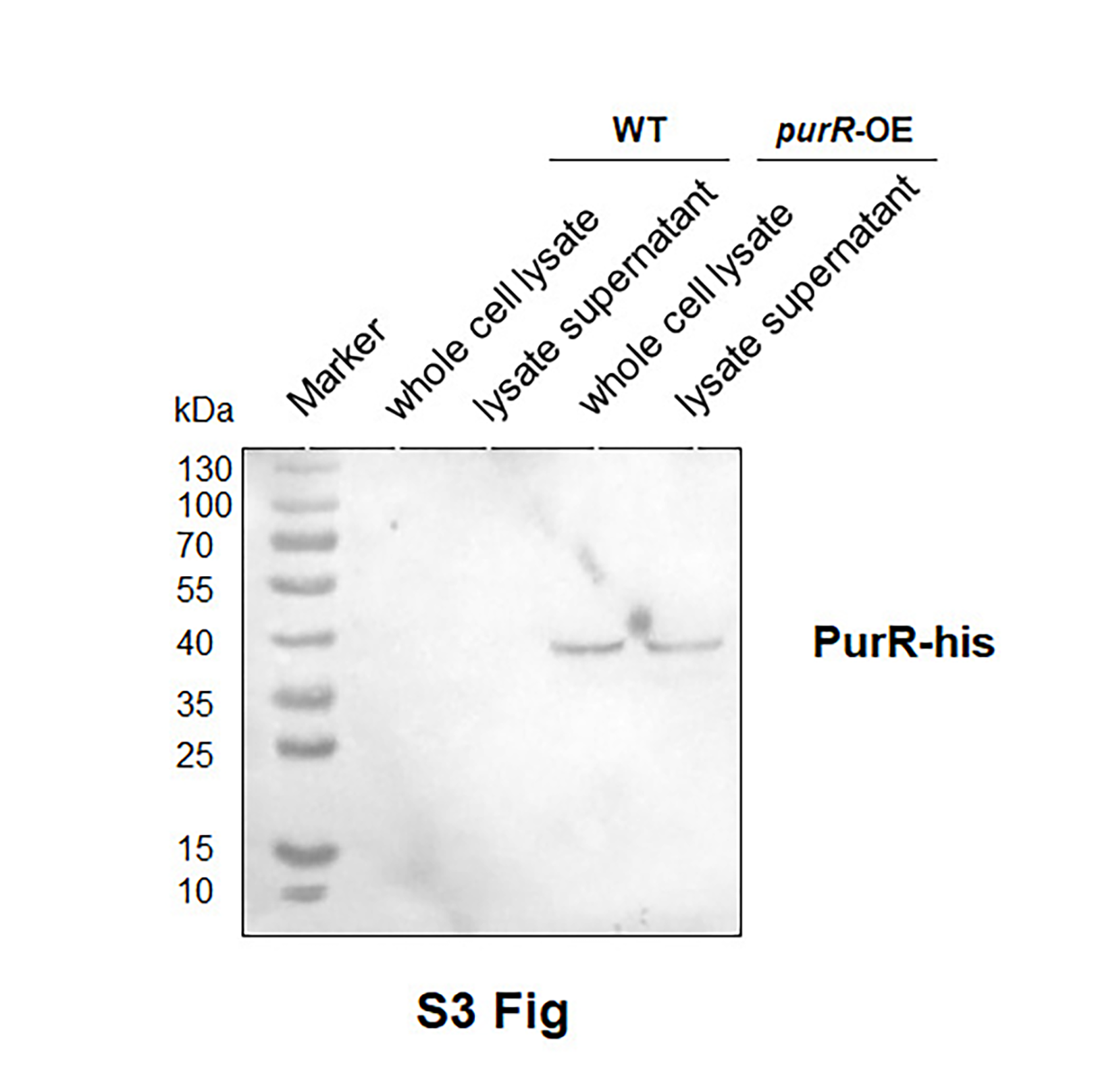

Supplement: S3 Fig — ExPEC strain PCN033 or PCN033 carrying pCDF-J23110-PurRhis plasmid (purR-OE) was grown to the mid-log phase in LB medium. Cells were harvested, and the whole cell lysate and lysate supernatant were prepared. The samples were subjected to SDS-PAGE followed by Western blotting analysis using an anti-His monoclonal antibody as the primary antibody. (TIF) [file ppat.1013889.s003.tif]
