# Supplementary material for: De novo nucleotide biosynthesis and its dynamic regulation are crucial for systemic infection by extraintestinal Escherichia coli
Source: PLoS Pathog. 2026 Jan 26;22(1):e1013889. doi: 10.1371/journal.ppat.1013889 (PMC12858057; doi:10.1371/journal.ppat.1013889)
Supplement: S1 Table — (DOCX) [file ppat.1013889.s004.docx]

**S1 Table. Bacterial strains used in this study**

| **Strain name** | **Description** | **Source** |
| --- | --- | --- |
| ExPEC PCN033 | Wild-type ExPEC clinical isolate | Lab stock |
| Δ*guaB* | *guaB* knockout mutant of ExPEC PCN033 | This study |
| Δ*purE* | *purE* knockout mutant of ExPEC PCN033 | This study |
| Δ*purR* | *purR* knockout mutant of ExPEC PCN033 | This study |
| Δ*purH* | *purH* knockout mutant of ExPEC PCN033 | This study |
| CΔ*guaB* | Complementary strain of Δ*guaB* | This study |
| CΔ*purE* | Complementary strain of Δ*purE* | This study |
| CΔ*purR* | Complementary strain of Δ*purR* | This study |
| CΔ*purH* | Complementary strain of Δ*purH* | This study |
| *purR*-OE | PurR overexpression strain (PCN033 strain carrying pCDF-J23110-purRhis) | This study |
| *E.coli* DH5α λPir | Cloning host | Lab stock |
| *E.coli* χ7213 | Conjugation donor strain | Lab stock |
| *E.coli* BL21 | Protein expression host | Vazyme Biotech Co., Ltd (Nanjing, China) |
| *E. coli* MC4100 | Commensal *E. coli* K-12 strain | Lab stock |
